# Sequential alterations in diffusion metrics as correlates of disease severity in amyotrophic lateral sclerosis

**DOI:** 10.1007/s00415-023-11582-9

**Published:** 2023-02-10

**Authors:** Hans-Peter Müller, Anna Behler, Maximilian Münch, Johannes Dorst, Albert C. Ludolph, Jan Kassubek

**Affiliations:** 1 Department of Neurology, University Hospital Ulm, Oberer Eselsberg 45, 89081 Ulm, Germany; 2 German Center for Neurodegenerative Diseases (DZNE), Ulm, Germany

**Keywords:** Amyotrophic lateral sclerosis, Diffusion tensor imaging, Motor neuron disease, Magnetic resonance imaging, Clinical score, ALS-FRS-R

## Abstract

**Background and objective:**

The neuropathology of amyotrophic lateral sclerosis (ALS) follows a regional distribution pattern in the brain with four stages. Using diffusion tensor imaging (DTI), this pattern can be translated into a tract-based staging scheme to assess cerebral progression in vivo. This study investigates the association between the sequential alteration pattern and disease severity in patients with ALS.

**Methods:**

DTI data of 325 patients with ALS and 130 healthy controls were analyzed in a tract of interest (TOI)-based approach. Patients were categorized according to their ALS-FRS-R scores into groups with declining functionality. The fractional anisotropy (FA) values in the tracts associated with neuropathological stages were group-wise compared with healthy controls.

**Results:**

The FA in the tracts associated with ALS stages showed a decrease which could be related to the disease severity stratification, i.e., at the group level, the lower the ALS-FRS-R of the categorized patient group, the higher was the effect size of the stage-related tract. In the patient group with the highest ALS-FRS-R, Cohen’s *d* showed a medium effect size in the corticospinal tract and small effect sizes in the other stage-related tracts. Overall, the lower the ALS-FRS-R of the categorized patient group the higher was the effect size of the comparison with healthy controls.

**Conclusion:**

The progression of white matter alterations across tracts according to the model of sequential tract involvement is associated with clinical disease severity in patients with ALS, suggesting the use of staging-based DTI as a technical marker for disease progression.

## Introduction

In amyotrophic lateral sclerosis (ALS), according to postmortem studies, four neuropathological stages of disease progression can be defined based on the regional distribution of the phosphorylated 43 kDa TAR DNA-binding protein (pTDP-43) in the brain [1, 2]. This neuropathological pattern can be translated to an in vivo staging concept for patients with ALS using diffusion tensor imaging (DTI), i.e., specifically, fractional anisotropy (FA) can be used to detect changes within the white matter to reveal the sequential involvement of specific tracts in ALS [3–5].

Among white matter alterations, the diffusion metrics of the corticospinal tract (CST), related to ALS stage 1, are particularly affected in ALS [6–8]. The FA decrease in the CST is associated with clinical severity cross-sectionally [9–11] and longitudinally [4, 12–14]. To assess the clinical severity and to monitor functional disability, the revised ALS Functional Rating Scale (ALS-FRS-R) [15] is commonly used and constitutes one of the standard primary endpoints in clinical trials [16]. In addition to the FA alterations in the CST, an association with the total ALS-FRS-R score was also shown for the DTI staging scheme at the individual level [3–5].

According to a hypothetical model [17], FA alterations in stage-associated tracts might occur together with worsening of clinical presentation as follows: the onset of the first symptoms is concurrent with the start of FA decrease in the CST. During the disease course, the FA in the CST further decreases, and FA alterations in corticopontine and corticorubral tracts (related to ALS stage 2) occur. Sequentially, the corticostriatal pathway (associated with ALS stage 3) and the proximal portion of the perforant path (related to ALS stage 4) also become involved in the FA alteration pattern during further disease progression. Thereby, the onset of alterations in the stage-associated tract systems may be associated with clinical milestones which might allow a translation of cerebral progression to clinical measures.

Owing to the heterogeneity of progression rates in ALS, the loss of functionality over a period of time can vary widely between patients. Therefore, the aim of this study was to investigate further the hypothesized model of sequential tract involvement in relation to the disease severity, i.e., loss of functionality. In addition to the previous studies showing stage-associated progression in ALS using DTI [4], the current study demonstrates the association of the DTI-based metric FA with the clinical presentation and severity (ALS-FRS-R including subscores).

## Methods

### Subjects and clinical characterization

The cross-sectional study cohort included 325 patients (61.5 ± 12.0 years, 198 male/126 female) with clinically definite or probable sporadic ALS, according to the revised version of the El Escorial World Federation of Neurology criteria [18] and 130 age- and sex-matched healthy controls (57.6 ± 12.1 years, 72 male/58 female). This cohort has been investigated in a previous study and is described in detail there [5]. All patients with ALS underwent standardized clinical-neurological and routine laboratory examinations. None of the patients had any history of other neurological or psychiatric disorders. The severity of patients' physical symptoms was measured with the ALS-FRS-R. The total ALS-FRS-R score was 40 ± 6 (mean ± standard deviation) and ranged from 17 to 47. In the current study, an ALS-FRS-R score at the time of MRI scanning was available in all 325 patients; these patients were categorized into ALS-FRS-R groups with decreasing total ALS-FRS-R in order to investigate their cross-sectional DTI scans by FA analysis in stage-related tracts. Accordingly, patients were subdivided into five groups based on their total ALS-FRS-R scores as follows: 74 patients (58.5 ± 10.3 years, 46 male/28 female) with an ALS-FRS-R score between 48 and 45, 92 patients (64.0 ± 9.4 years, 55 male/37 female) with an ALS-FRS-R score between 44 and 41, 81 patients (60.3 ± 13.7 years, 53 male/27 female) with an ALS-FRS-R score between 40 and 37, 42 patients (65.4 ± 11.1 years, 21 male/21 female) with an ALS-FRS-R score between 36 and 33, and 35 patients (59.3 ± 15.1 years, 23 male/12 female) with an ALS-FRS-R score of 32 or less. All subjects gave written consent for the MRI protocol according to institutional guidelines. The study was approved by the Ethical Committee of the University of Ulm, Germany (reference # 19/12).

### MRI acquisition and DTI analysis

DTI data were acquired either on a 1.5 T scanner (248 patients and 80 healthy controls) or a 3.0 T scanner (77 patients and 50 healthy controls). Data processing was performed with the DTI analysis software ‘Tensor Imaging and Fiber Tracking’ (TIFT) [19]. The data analysis cascade followed an established protocol [5, 10]; in short, DTI data sets underwent quality control [20] and were normalized iteratively to the Montreal Neurological Institute (MNI) stereotaxic standard space [21]. FA maps were then calculated from each data set, smoothed with a spatial Gaussian filter of 8 mm full width at half maximum, and corrected for different scanners and different acquisition protocols [10, 22]. By a seed-to-target tract of interest (TOI)-based approach, the following brain structures associated with the ALS staging system [3–5] were identified: the CST (according to ALS stage 1), the corticorubral and corticopontine tracts (according to ALS stage 2), the corticostriatal pathway (according to ALS stage 3), and the proximal portion of the perforant path (according to ALS stage 4). FA values of corticopontine and corticorubral tracts were averaged as both were associated to stage 2. Fiber tracking was a deterministic streamline tracking approach, and the technique of tract-wise fractional anisotropy statistics (TFAS) [23] was used to determine the mean FA values of the tracts; bihemispheric FA values were averaged and corrected for age [24].

For each tract, the FA values of patients with ALS and healthy controls were then tract-wise *z*-transformed to the mean and standard deviation of the healthy controls sample. For comparisons at the group level, *t* tests were performed and the effect size was calculated as Cohen’s *d* which can be interpreted as very small (*d* = 0.1), small (*d* = 0.2), medium (*d* = 0.5), large (*d* = 0.8), very large (*d* = 1.2), or huge effect (*d* = 2.0) [25]. Significance was defined at *α* = 0.05, Bonferroni-corrected for multiple comparisons.

In order to test for associations between the FA values of the CST with the limb subscore of the ALS-FRS-R as well as between the FA values of stage 2-related tracts (corticopontine and corticorubral tracts) with the bulbar subscore of the ALS-FRS-R, respectively, correlations were calculated by Spearman’s rank correlation.

## Results

**Fig. 1:**
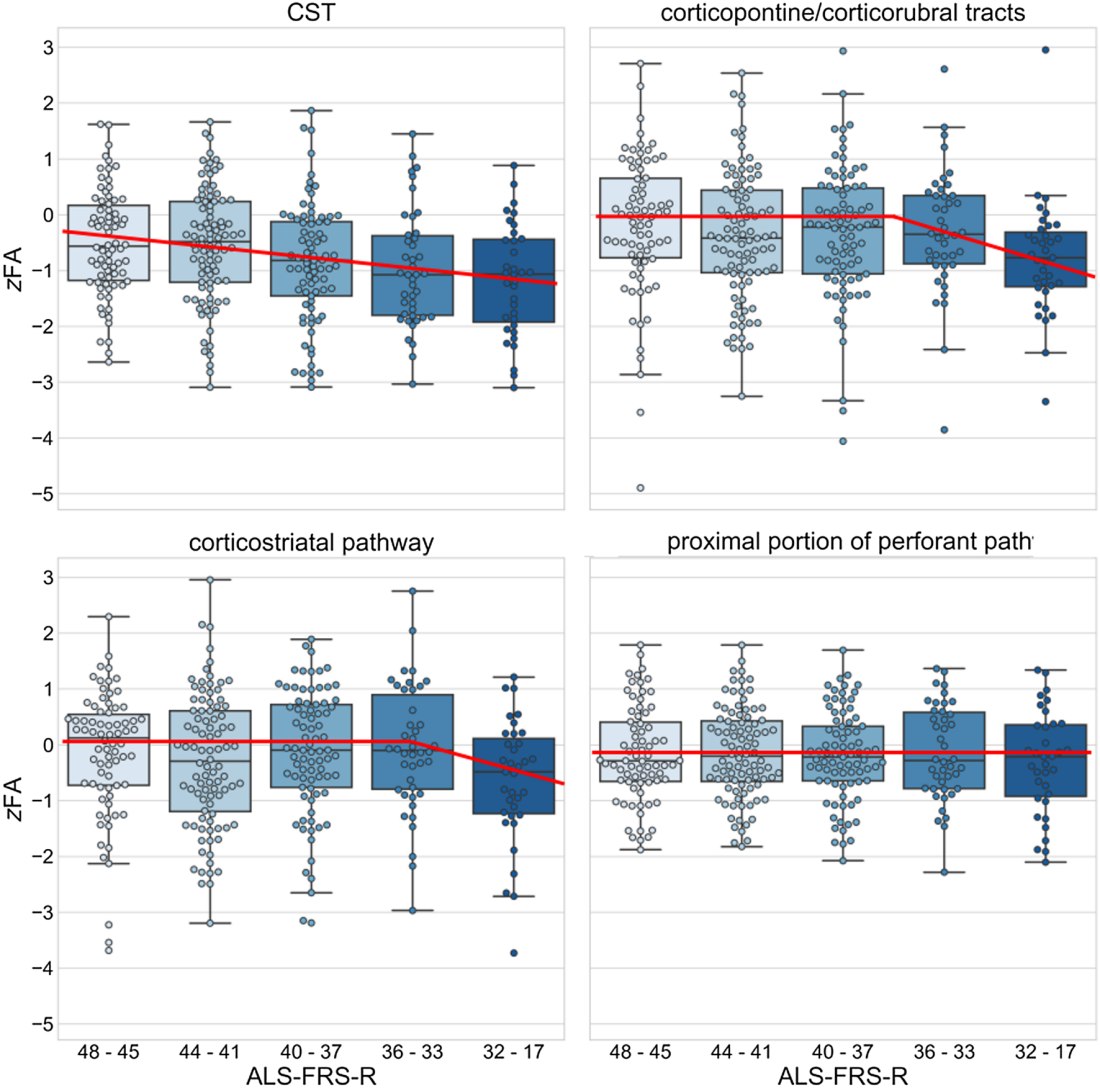
Boxplots of *z*-standardized fractional anisotropy (*z*FA) values in stage-associated tracts in amyotrophic lateral sclerosis (ALS) across patient groups with different total ALS-FRS-R scores. With increasing clinical severity, the FA values in the tracts decrease according to the diffusion tensor imaging staging scheme (red lines as representations of a hypothetical model [17]). *ALS-FRS-R* ALS functional rating scale revised

The effect sizes (Cohen’s *d*) and *p* values for tract-wise comparisons of all ALS-FRS-R data with healthy controls are provided in Table 1. The distributions of tract-specific FA values of patients after categorization according to their ALS-FRS-R scores are shown in Fig. 1.

**Table 1:** Results of the statistical comparison of the tract-based fractional anisotropy of patients with amyotrophic lateral sclerosis (ALS) in different ALS-FRS-R categories with the healthy control group: Cohen’s *d* and the *p* value from Student’s *t* test with a significance level of 0.0025, corrected for multiple testing

ALS-FRS-R	48–45		44–41		40–37		36–33		< 32	
*N*	74		92		81		42		35	
	*d*	*p*	*d*	*p*	*d*	*p*	*d*	*p*	*d*	*p*
ALS stage 1	0.51	0.0005	0.55	< 0.0001	0.84	< 0.0001	0.96	< 0.0001	1.18	< 0.0001
ALS stage 2	0.18	> 0.05	0.36	0.0110	0.30	0.0416	0.28	> 0.05	0.79	0.0002
ALS stage 3	0.14	> 0.05	0.27	> 0.05	0.13	> 0.05	0.06	> 0.05	0.62	0.0029
ALS stage 4	0.18	> 0.05	0.17	> 0.05	0.21	> 0.05	0.15	> 0.05	0.32	> 0.05

*ALS-FRS-R* revised ALS functional rating scale, *ALS stage 1* corticospinal tract, *ALS stage 2* corticorubral/corticopontine tract, *ALS stage 3* corticostriatal pathway, *ALS stage 4* proximal portion of the perforant path

At the group level, the FA in the CST was significantly decreased for patients in all five ALS-FRS-R categories as compared to healthy controls. In the group of patients with an ALS-FRS-R between 48 and 45, the effect size was medium and was the higher the lower the ALS-FRS-R scores were, resulting in a large effect size in patients with an ALS-FRS-R below 32. Overall, the FA in the tracts related to ALS stages 2–4 showed also a decrease; more specifically, at the group level, the lower the ALS-FRS-R of the categorized patient group the higher was the effect size of the comparison with healthy controls. The exception is the group of patients with an ALS-FRS-R of 33–36: herein the Cohen’s *d* values for stage 2–4 are lower as compared to the neighbored ALS-FRS-R groups.

The tract-specific FA of the CST (stage 1-related tract) showed a significant association with the limb subscore of ALS-FRS-R (*C* = 0.15; *p* = 0.012), and the tract-specific FA of the stage 2-related tracts (corticopontine and corticorubral tracts) showed a significant association with the bulbar subscore of ALS-FRS-R (*C* = 0.20; *p* = 0.0011).

In the whole ALS sample of 325 subjects, 28 had a prominent upper motor-neuron prevalence and 90 had a prominent lower motor-neuron prevalence. These subgroups were analysed separately, but no significant differences in tract-based FA values between those two subgroups were detected.

## Discussion

The FA in the tracts related to the neuropathological stages of ALS was analyzed for data of patients with declining functionality according to ALS-FRS-R scores. This analysis demonstrated a straightforward association between the clinical ALS-FRS-R and the TOI-based in vivo sequential staging scheme. The FA in the CST was significantly reduced in the patient group with mild functional loss (i.e., a total ALS-FRS-R between 48 and 45), in line with previously published studies [10, 26] and the association of the CST with neuropathological stage 1 [3, 4]. Altogether, the FA in the tracts related to ALS stages 1–4 showed a decrease related to function impairment, i.e., at the group level, the lower the ALS-FRS-R of the categorized patient group, the higher the effect size was. The results support the hypothesis of sequential involvement of tracts/neural pathways in the course of disease progression [17]. In a previous longitudinal study, the sequential onset of white matter changes corresponding to an increase in the DTI stage over time could already be shown at the individual patient level [4]. In the present analysis of the FA of the stage-associated tract systems across disease severity levels, it could be demonstrated at the group level that the beginning loss of functionality is associated with a pattern of tract-specific alterations corresponding to DTI stage 1. Altogether, the main result of this study was that at the group level with decreasing ALS-FRS-R, the tracts that are related to the specific ALS stages become more and more involved that way supporting the hypothetic model of a sequential involvement of white matter neuronal tracts during the course of the disease [17]. The patients with ALS-FRS-R of 33–36 show low Cohen’s *d* values for stages 2–4, not in accordance with the sequential pattern of increasing Cohen’s *d* values for increasing ALS-FRS-R. However, given that this is the ALS-FRS-R subgroup with a limited subject number (*N* = 42), statistical effects are apparently the main factor. This constitutes a limitation for the interpretation of the results pattern which implies increasing Cohen's d values for increasing ALS-FRS-R; nevertheless, as an overall result, increasing Cohen’s *d* values for increasing ALS-FRS-R could be postulated. Significant correlations of tract-specific FA with limb and bulbar subscores of ALS-FRS-R, respectively, allow for an interpretation that the CST is particularly associated with the limb affectation and stage 2-related tracts are associated with the bulbar symptoms. Here, the study demonstrates the ability of TOI-based DTI to map the disease-related stages of ALS in vivo so that MRI has a differential part than fluid markers like neurofilament light chain which correlates with disease progression rate and is negatively associated with survival, thus providing prognostic information [27].

However, the current findings should be considered in the context of some limitations. First, to detect a detailed longitudinal association between disease severity and cerebral stages at the individual level, multiple, closely scheduled DTI scans over a period of time with associated functional decline would be necessary. Currently, retrospective (multicenter) data sets [4, 26] contain predominantly patients with only one or two follow-up measurements, with only mild to moderate deterioration in functionality between the first and last scan. Subsequent neuroimaging studies focusing on mapping clinical progression to spread of cerebral alterations would be necessary. Owing to the limited availability of patients with an ALS-FRS-R of ≤ 32 for MRI scanning, a small-scale analysis could not be performed for a severe loss of functionality. This may have prevented alterations from being observed for tracts associated with stage 4 at the group level, i.e., the proximal portion of the perforant path. Third, the in vivo findings of cerebral involvement have not been confirmed ex vivo in our patients. Fourth, due to the ex post facto character of this study, the number of patients differed between groups, that way influencing the statistical significance (*p* values, Table 1). To overcome this challenge, this study focused on Cohen’s *d* to report the effect size in each ALS-related tract for each patient group with different ALS-FRS-R. Fifth, the clinical severity was represented by the total ALS-FRS-R score which might not reflect all clinical deficits and neuropsychological dysfunctions of the patients. In addition, the ALS-associated neuropathological changes in the brain [2] can be reflected to only a limited extent by assessing functionality with the ALS-FRS-R, given that many clinical dysfunctions are determined by second motor neuron degeneration. As the total ALS-FRS-R includes various subscores, identical total scores of patients might not be completely comparable concerning disease status [28, 29]; however, we considered ALS-FRS-R scores to be the best clinical measure of functionality grosso modo.

In summary, clinical worse state was shown to be associated with regional spread of white matter alterations as assessed by the DTI-based staging scheme in patients with ALS. Thus, in accordance with the a priori hypothesis, the association of cerebral disease progression seems to go along with clinical progression as measured by the ALS-FRS-R. These findings indicate that TOI-based FA mapping could provide an additional imaging-based marker for ALS progression which might be useful in a setting of clinical trials to potentially add to the assessment of future disease-modifying effects in ALS.

## Data Availability

Data are available upon reasonable request. Reasonable data sharing requests require a formal data sharing agreement. Data sharing agreements must include details on how the data will be stored, who will have access to the data and intended use of the data, and agreements as to the allocation of intellectual property.
